# Fecal DNA testing for Colorectal Cancer Screening: the ColoSure™ test

**DOI:** 10.1371/currents.RRN1220

**Published:** 2011-03-22

**Authors:** Renée M. Ned, Stephanie Melillo, Michael Marrone

**Affiliations:** Office of Public Health Genomics, Centers for Disease Control and Prevention

## Abstract

Colorectal cancer is the third most common cancer and the second leading cause of cancer-related deaths in the United States. Screening has been shown to be effective in reducing colorectal cancer incidence and mortality. Colonoscopy, sigmoidoscopy, and fecal occult blood tests are all recommended screening tests that have widespread availability. Nevertheless, many people do not receive the evidence-based recommended screening for colorectal cancer. Additional stool-based methods have been developed that offer more options for colorectal cancer screening, including a variety of fecal DNA tests. The only fecal DNA test that is currently available commercially in the United States is ColoSure(TM), which is marketed as a non-invasive test that detects an epigenetic marker (methylated vimentin) associated with colorectal cancer and pre-cancerous adenomas. We examined the published literature on the analytic validity, clinical validity, and clinical utility of ColoSure and we briefly summarized the current colorectal cancer screening guidelines regarding fecal DNA testing. We also addressed the public health implications of the test and contextual issues surrounding the integration of fecal DNA testing into current colorectal cancer screening strategies. The primary goal was to provide a basic overview of ColoSure and identify gaps in knowledge and evidence that affect the recommendation and adoption of the test in colorectal cancer screening strategies.

## **Application: Screening**

## **Background**

**Colorectal Cancer (CRC) screening**

Screening by colonoscopy, sigmoidoscopy and fecal occult blood testing has been shown to prevent colorectal cancer (CRC) and to reduce mortality through the detection and removal of pre-cancerous lesions and through the detection of CRC in its early stages [1] [2]. Indeed, CRC incidence and mortality have been decreasing since 1985 [2] [3]. Research suggests that CRC screening may be responsible for approximately half of the declines [2]. However, uptake of CRC screening recommendations in the U.S. is not optimal. In 2008, only about 62% of men and women aged 50-75 years reported getting the most commonly recommended CRC screening tests, a percentage that varied from 49-75% among states [4].    

Several tests are available to identify colorectal cancer and pre-cancerous polyps in asymptomatic individuals. Colonoscopy visually inspects the interior walls of the entire rectum and colon.  Performance characteristics (such as sensitivity and specificity) of new tests are commonly evaluated in comparison with colonoscopy [1] [5]. Flexible sigmoidoscopy involves a more limited visual inspection of the distal colon and rectum. Fecal occult blood tests (FOBTs), which include conventional guaiac FOBT, high-sensitivity guaiac FOBT, and fecal immunochemical tests (FITs), chemically detect small amounts of fecal blood (which can originate from pre-cancerous and cancerous colorectal lesions).  CT colonography (i.e., virtual colonoscopy) and double-contrast barium enema (DCBE) are additional tests, offering enhanced x-ray images of the interior rectum and colon to aid in detecting abnormalities.  

Fecal (stool) DNA tests have been under continuous development over the past several years. These tests are designed to detect in stool samples any number of DNA markers shown to be associated with CRC. ColoSure™ is the latest example of a clinically available stool DNA test.  

**Clinical Scenario**

The clinical scenario for fecal DNA testing in general is most often presented as colorectal cancer screening in average-risk individuals. 

A technical brochure for ColoSure [6] states that:

“ColoSure is not intended to replace a colonoscopy in those patients who are willing and able to undergo the procedure. Additionally, while it may be used adjunctively or in patients noncompliant with screening recommendations, it is not a screening tool for individuals at increased risk for developing disease.”

**Test Description**

ColoSure™ (Laboratory Corporation of America, http://www.labcorp.com) is currently the only commercially or clinically available fecal DNA test marketed for CRC screening in the U.S. The at-home test requires that patients collect and mail one whole stool sample. The test was developed by the Laboratory Corporation of America (LabCorp), which required licensing intellectual property from Exact Sciences Corporation (www.exactsciences.com). As a laboratory-developed (“home-brewed”) test, ColoSure is not subject to regulation by the U.S. Food and Drug Administration (FDA) and has not obtained FDA clearance or approval.

What is the theory behind stool DNA testing?  Colorectal cancer cells, which are shed into the feces, are known to have several genetic alterations which offer an array of molecular targets for DNA-based stool testing for both pre-cancerous and cancerous lesions [7] [8]. Consequently, fecal DNA has been explored for its potential as a non-invasive CRC screening methodology.

ColoSure is a single-marker test that detects methylation of the vimentin gene. Increased DNA methylation in the promoter region of genes is an epigenetic change that is common in human cancers, including colorectal cancer [9] [10]. Vimentin is a protein characteristically expressed in cells of mesenchymal origin, such as fibroblasts, macrophages, smooth muscle cells, and endothelial cells. Studies have demonstrated that the vimentin gene is not (or rarely) methylated in normal colonic epithelial cells, but is methylated in colorectal cancer and adenomas [11] [12] [13]. Aberrant methylation of vimentin has been detected in 53-83% of colorectal cancer tissue, 50-84% of adenoma samples, and 0-11% of normal colon tissue samples [11] [12] [13] [14] [15], though one preliminary study detected methylated vimentin in 29% of normal colon tissue [16]. 

ColoSure requires a prescription for testing. It is currently available from two sources: LabCorp [17] and from DNA Direct’s Genomic Medicine Institutes (which only offers referrals to physicians who can prescribe the test) [18] [19].   

**Public Health Importance**

Colorectal cancer (CRC) is currently the third leading cancer diagnosed in the United States, where the lifetime risk is approximately 5% in the general population [3] [7]. According to the U.S. Cancer Statistics, ~143,000 cases of CRC occurred in the U.S. in 2007 [20]. CRC is also the second leading cause of cancer-related deaths in the United States, with approximately 53,000 deaths occurring in 2007 [20]. Approximately half of colorectal cancers are diagnosed at a late stage, when survival is poorer [4].  

The most effective way of reducing the risk of developing CRC and of reducing CRC mortality is early detection and removal of pre-cancerous or cancerous lesions. It is thought that the natural history of CRC development takes between 10 and 20 years, offering an excellent opportunity for early intervention [1] [21].  

Three types of tests (colonoscopy, flexible sigmoidoscopy, and fecal occult blood tests) are currently recommended as evidence-based CRC screening options by the U.S. Preventive Services Task Force [1]. However, only a modest percentage of adults meet the recommended CRC screening guidelines [4].  

Stool-based DNA tests are suggested by some experts as another option for CRC screening. However, these tests are under rapid development and research to establish analytic validity, clinical validity, and clinical utility within the general (average-risk) population is needed before any fecal DNA test can be integrated into current CRC screening strategies. We now examine these factors for the ColoSure test based on the current literature. 

**Published Reviews, Recommendations and Guidelines (see Table 1 below)**

**Important Note:  The following groups considered fecal DNA testing in general, but largely based their recommendations and guidelines on published research relevant to stool DNA tests that are no longer commercially available.**

**Systematic evidence reviews**

The Agency for Healthcare Research and Quality (AHRQ) commissioned an evidence report/technology assessment on enhancing the use and quality of CRC screening [22] [23]. They found no reliable data among the included studies concerning the trends in use or quality (evidence of misuse, overuse, or underuse) of fecal DNA testing.  

A systematic evidence review was performed that guided the current recommendations on CRC screening by the U.S. Preventive Services Task Force (USPSTF) [5] [24] (see subsection below). 

**

**Recommendations by independent group**

Fecal DNA testing was considered by the USPSTF in its most recent recommendation statement on CRC screening (see **Table 1** below). The USPSTF found insufficient evidence to evaluate the benefits and harms of this kind of testing as a screening modality for CRC (I statement) [1].          

**Guidelines by professional** **groups (in order by year of publication)**

A Joint Guideline was published in 2008 by the American Cancer Society, the U.S. Multi-Society Task Force on Colorectal Cancer, and the American College of Radiology (ACS-USMSTF-ACR) [25]. Stool DNA testing in general was recommended for those aged 50 years or older, but the testing interval could not be determined. In 2009, the American College of Gastroenterology (ACG) published CRC screening guidelines, in which a weak recommendation (Grade 2B) was made for stool DNA testing every 3 years for persons 50 years of age or older [26]. The American Academy of Family Physicians (AAFP) [27] published recommendations in 2010, which deferred to the analysis and findings of the USPSTF. Also in 2010, the guidelines of the National Comprehensive Cancer Network (NCCN) [28] stated that: 1) stool DNA testing is not currently considered a first-line screening test except in specific circumstances; and, that 2) the testing interval is uncertain.  

**Health Plan/Payer policies**

CRC screening guidelines have been issued by Kaiser Permanente [29], Aetna, Inc. [30], and United Healthcare Group [31] [32], all of which describe fecal DNA screening as experimental and not recommended for use.  

A summary of all mentioned recommendations and guidelines appear in Table 1 below.    

**Table 1. Routine Colorectal Cancer Screening Guidelines and Recommendations for average-risk adults.**

**Table:** 

	**Colonoscopy**	**FSlG**	**FOBT**	**FIT**	**DCBE**	**CTC**	**sDNA test**
**USPSTF 2008 **	Every 10y, Ages 50-75	Every 5y, Ages 50-75^1^	Annually, Ages 50-75	Annually, Ages 50-75	NM	Insufficient evidence	Insufficient Evidence
**ACS-USMSTF-ACR 2008**	Every 10y, Age >50y	Every 5y, Age >50y	Annually, Age >50y	Annually, Age >50y	Every 5y, Age >50y	Every 5y, Age >50y	Recommended for age > 50y, but at uncertain interval
**ACG 2009 **	Every 10y, Age >50y	Every 5-10y, Age >50y	Annually, Age >50y	Annually, Age >50y	NM	Every 5y, Age >50y	Every 3y, Age >50y
**AAFP 2010 **	Same as USPSTF	Same as USPSTF	Same as USPSTF	NM	NM	Same as USPSTF	Same as USPSTF
**NCCN 2010 **	Every 10y, Age >50y	Every 5y, Age >50y	Annually, Age >50y	Annually, Age >50y	NM	Every 5y, Age >50y	Not a first-line screening test except in specific circumstances, uncertain interval
**Kaiser **	Average risk adults	Average risk adults	Average risk adults	Average risk adults	NR	NR	NR
**Aetna **	Every 10y, Age >50y	Every 5y, Age >50y	Annually, Age >50y	Annually, Age >50y	Every 5y, Age >50y	Designated as experimental and investigational	Designated as experimental and investigational
**United Healthcare Group **	Every 10y, Age >50y	Every 5y, Age >50y	Annually, Age >50y	Annually, Age >50y	Every 5y, Age >50y	NM	Designated as experimental and investigational

CTC = CT colonoscopy; DCBE = double-contrast barium enema; FIT = fecal immunochemical test; FOBT = fecal occult blood test; FSIG = flexible sigmoidoscopy; sDNA = stool DNA.

NM = not mentioned; NR = Not Recommended.   ^1^ In combination with high-sensitivity FOBT every 3 years.

**Evidence Overview**

**We performed literature searches (through PubMed and Ovid MEDLINE) that included search terms such as “vimentin”, “fecal DNA”, and “colorectal cancer”.**

***Analytic Validity*: **Test accuracy and reliability in measuring methylated vimentin (analytic sensitivity and specificity).  

- We found no published data on the analytic sensitivity or specificity of the ColoSure test for methylated vimentin. 
- The amount (total and relative) of methylated vimentin in stool samples can vary widely in patients with adenoma or colorectal cancer [33].
- The methylation-specific PCR (MSP) assay used in the ColoSure test [6] may not have adequate sensitivity for detecting methylated vimentin. Researchers have shown an inability to detect methylated vimentin using MSP in CRC patient stool samples (n = 8) that contained low concentrations of human DNA. However, these researchers demonstrated that methyl-binding domain protein enrichment prior to MSP increased assay sensitivity [34]. In addition, a new technology— methyl-BEAMing— has been developed that enhances the overall technical sensitivity for detecting methylated vimentin by at least 62-fold relative to MSP [33]. 

Summary: the analytic validity of the ColoSure test could not be determined from the identified research. 

***Clinical Validity*: **Test accuracy and reliability in detecting colorectal cancer or adenomas (clinical sensitivity and specificity; predictive value).

- In general, one potential advantage of DNA-based stool tests over FOBTs is the continuous exfoliation of colorectal cells into the feces (as opposed to occult bleeding, which is intermittent).  This finding possibly increases the sensitivity of stool DNA tests [8] [35]. 
- Six studies relevant to the clinical validity of the ColoSure test were identified [11] [14] [33] [36] [37] [38]. These studies are summarized below in Table 2, in which research findings on methylated vimentin as a stand-alone marker are highlighted.
- All 6 studies were case-control in design, having selected patients known to have CRC confirmed by colonoscopy compared with controls who were negative for CRC after colonoscopy. None of these analyses were conducted prospectively or in a general screening population (ages 50-75 yrs, average CRC risk). It is, therefore, important to interpret these observational data with caution, as some methodologists report that case-control studies tend to overestimate screening or diagnostic accuracy due to design-related bias [39] [40] [41].

**Table 2. Summary of the published case-control studies relevant to ColoSure that reported measures of clinical validity using fecal DNA testing in selected populations ** 

**Table:** 

		**Sensitivity**		**Specificity**
**Study**	**Marker(s)**	**CRC**	**Adenoma** ^**1**^	

**Chen 2005 [11]**	Methylated *vimentin*	46% (43/94)	--	90% (178/198)
**Itzkowitz 2007 [38]**	Methylated *vimentin*	73% (29/40)^2^	--	87% (106/122)^2^
(Phase 1a)	DY	65% (26/40)^2^	--	93% (113/122)^2^	
	Methylated *vimentin* or DY	88% (35/40)^2^	--	82% (100/122)^2^	
**Itzkowitz 2008 [37]**				
(Phase 1b)	Methylated *vimentin*	81% (34/42)^2^	--	82% (198/241)^2^	
	DY	60% (25/42)^2^	--	85% (205/241)^2^	
	Methylated *vimentin* or DY	86% (36/42)^2^	--	73% (176/241)^2^	
(Combined Data)	Methylated *vimentin*	77% (63/82)^2,3^	--	83% ( 301/363)^2,3^	
	DY	48% (39/82)^2,3^	--	96% (348/363)^2,3^	
	Methylated *vimentin* or DY	83% (68/82)^2,3^	--	82% (298/363)^2,3^	
**Ahlquist 2008 [14]**	Test SDT-2 (point mutations on *K-ras*, scanned mutator cluster region of *APC*, methylated *vimentin*)	58% (7/12)^2^	46% (47/103)^2^	Not calculated	
**Baek 2009 [36]**	*mMLH1*	30% (18/60)	12% (6/52)	100% (37/37)^4^	
	Methylated *vimentin*	38% (23/60)	15% (8/52)	100% (37/37)^4^	
	MGMT	52% (31/60)	37% (19/52)	86% (32/37)	
	All three markers (combined)	75% (45/60)	60% (31/52)	86% (32/37)	
**Li 2009 [33]**	Methylated *vimentin*	41% (9/22)	45% (9/20)	95% (36/38)	

DY = refers to a specific test for DNA integrity.

-- Not measured.

^1^ Refers to adenomas ≥ 1 cm.

^2^ We calculated the numerator using data presented in the article.

^3^ In the study, sensitivity and specificity were calculated using optimal cutpoints based on the combined dataset (Phases 1a + 1b).

^4^ We calculated specificity using data presented in the article.    

- Due to the processes for sample collection, sample preparation, and laboratory analysis, the most relevant findings on ColoSure appear to be contained in the two Itzkowitz, *et al. *reports [37] [38]. In these studies, a second assay (for DNA integrity) was also examined alone and in combination with methylated vimentin. The combined findings from both phases of the study [37] suggest a sensitivity for CRC of 77% and a specificity of 83% for methylated vimentin. However, informational materials for ColoSure also reference internal LabCorp data, which, combined with the Itzkowitz, *et al.* studies, suggest that ColoSure has 72-77% sensitivity and 83-94% specificity for CRC [6] [17]. 
- Itzkowitz, *et al.* did not specifically enroll patients with adenomas in their study populations [37] [38], so the clinical validity of ColoSure for pre-cancerous lesions is unclear.
- Using a more advanced technical method for detecting methylated vimentin, Li, *et al.* reported sensitivities of 45% and 41% for detecting adenomas and colorectal cancer, respectively, with ~95% specificity [33]. This research demonstrates that more sensitive methods of methylated vimentin detection (such as that in [33] [34]) would likely affect the clinical validity of ColoSure. Of note, Exact Sciences is in the process of developing more sensitive methods to detect methylated vimentin [42].
- It is unclear how fecal DNA screening using methylated vimentin compares to other established CRC screening tests. A pre-commercial version of the first-generation PreGen-Plus fecal DNA test was directly compared to a guaiac FOBT in a large multi-center study of asymptomatic persons [43], but no research has been published directly comparing ColoSure to other CRC screening methods. The SDT-2 test (which contains methylated vimentin) has been compared to two guaiac tests [14].

Summary:  the clinical validity of methylated vimentin as a biomarker for CRC screening remains to be determined in a general (average-risk) screening population. This is re-iterated in the LabCorp technical review for the ColoSure test [6], which states that: “The detection rates for general population screening have not been determined.”  

***Clinical Utility*: **Net benefit of test in improving health outcomes.

- The clinical utility of ColoSure for CRC screening has not been established through randomized controlled trials of CRC incidence or mortality outcomes. One ongoing prospective cohort study [NCT01270360] is examining the performance characteristics of both blood and/or stool based molecular DNA markers in identifying CRC in patients with positive FOBT, though it is unclear exactly which DNA markers are being tested. The study also aims to determine the cost-effectiveness of adding fecal DNA testing to the screening algorithm for patients with positive FOBT prior to colonoscopy. 
- ColoSure specifically has not been recommended by independent groups or professional organizations [1] [25] [26] [27] [28] to replace colonoscopy in any patient, regardless of whether they are willing and able to undergo the procedure and regardless of CRC risk level.
- From the patient’s perspective, stool DNA testing in general may have some advantages over colonoscopy for CRC screening since it: is non-invasive; does not require a formal health care visit; does not require dietary or medication restrictions, bowel preparation, or sedation; and does not require hours of time for testing and recovery, thus alleviating the need to take leave from normal activities (such as a job). 
- From the patient’s perspective, DNA-based stool tests may offer some advantages over FOBT, which requires multiple stool smears as well as some pre-test dietary and medication restrictions (which are necessary for guaiac-based testing). However, ColoSure does require handling a minimum 36 g sample of stool, which may be less acceptable than handling stool smears.
- Some studies have noted high patient satisfaction with fecal DNA testing or a patient preference for stool DNA testing over colonoscopy, though colonoscopy was perceived as the more accurate test [38] [44] [45]. However, other populations surveyed had a higher preference for colonoscopy than for fecal DNA testing [46].
- There is potential for the improvement in health outcomes if more people are willing to undergo fecal DNA testing compared to a screening colonoscopy or other invasive test methods, thereby increasing the percentage of adults who undergo CRC screening. In addition, the USPSTF notes that the chief benefit of less invasive screening tests (assuming they have adequate clinical sensitivity and specificity) is that they may reduce the number of colonoscopies required, since colonoscopies have risks of their own [1]. However, there are a few issues related to these ideas that need to be addressed:

- There is an uncertain disease detection benefit, unless fecal DNA is at least as sensitive as FOBTs [5] [24] [47] for pre-cancerous and cancerous lesions;
- Current research suggests that fecal DNA tests have poorer specificity than FOBT (especially guaiac-based or FIT) [5] [24] [47], which would lead to unnecessary colonoscopies due to a higher number of false positives;
- There is no research available to determine re-screening intervals for stool DNA testing;
- In general, fecal DNA testing may not be cost-effective when compared to other CRC screening tests [48];
- Patients may not comply with recommendations for frequent (e.g., annual or biennial) screening intervals. Indeed, longitudinal data have shown less than 50% adherence with screening frequency recommendations for stool-based tests such as FOBT [49];
- There may also be poor follow-up (e.g., colonoscopy) after a positive result on a fecal DNA test, as has been shown for FOBT [22].

Summary: the clinical utility of ColoSure (or methylated vimentin in general) in an average-risk screening population could not be determined from the identified research. 

**Final important note:**

Fecal DNA tests are under rapid development. Exact Sciences Corporation has developed several approaches to fecal DNA testing for colorectal cancer screening over the past few years. Previous tests were replaced sequentially with newer versions, which differed in laboratory methodology or tested for a different panel of DNA markers. The current ColoSure test is a replacement of a version of the PreGen-Plus™ test (Laboratory Corporation of America), which has been discontinued. Exact Sciences recently reported results from a validation study of its newest stool-based DNA test for colorectal cancer screening, named Cologuard™. The panel that was presented included methylated vimentin as one of the tested markers [42] [50] [51]. Exact Science is currently funding a case-only study [NCT01260168] to determine the sensitivity of this new multi-marker DNA panel in CRC cases. The company is planning to pursue FDA approval for Cologuard in 2012 [50]. These developments likely mean that ColoSure will be replaced in the future by this, or other, tests.  

**Concluding remarks:**

In order to consider integrating fecal DNA testing into current CRC screening strategies, additional research is needed to establish analytic validity, clinical validity, and clinical utility within the general (average-risk) population. The estimates of DNA marker sensitivity and specificity found from small case-control studies should not be extrapolated to make any estimates of the performance of methylated vimentin or ColoSure in the general population. 

In addition, the ongoing development and refinement of stool DNA tests presents some difficulty for the integration of these tests as a CRC screening approach. Currently, only one fecal DNA test is commercially available in the U.S., a test that will likely be replaced by a newer version for which FDA approval will be sought.

Other critical matters must also be addressed, including the determination of cost-effectiveness, optimal testing intervals, and strategies for the follow-up evaluation of patients who test positive on a fecal DNA test. Moreover, the willingness of individuals from the general population to adopt fecal DNA test protocols and future screening recommendations is a vital consideration. All of these factors will be crucial in affecting the impact of fecal DNA testing on the overall CRC screening paradigm and on colorectal cancer incidence and mortality.  

**Links (not referenced above)**

- Online Mendelian Inheritance in Men (OMIM) entry on colorectal cancer: http://www.ncbi.nlm.nih.gov/omim/114500
- CDC webpage: Colorectal Cancer Screening
- National Cancer Institute: Colorectal Cancer Screening Fact Sheet

Last updated: March 14, 2011     

**Acknowledgments**

The authors would like to thank the following individuals for invaluable input and guidance on the content of this manuscript: Dave Dotson, Ralph Coates and Katie Kolor (Office of Public Health Genomics, CDC); Lisa Richardson and Djenaba Joseph (Division of Cancer Prevention and Control, National Center for Chronic Disease Prevention and Health Promotion, CDC); and Evelyn Whitlock, Beth Webber, and their colleagues (Kaiser Permanente Center for Health Research).   

**Funding information**

This work was funded by the Office of Public Health Genomics, Centers for Disease Control and Prevention.  

**Competing interests**

The authors have declared that no competing interests exist.  

**Disclaimers**

The findings and conclusions in this report are those of the authors and do not necessarily represent the views of the Centers for Disease Control and Prevention (CDC).  

The information provided in this manuscript does not constitute an endorsement of ColoSure(TM) or of any fecal DNA test by the CDC nor the Department of Health and Human Services (DHHS) of the U.S. government. No endorsement should be inferred.  

The CDC does not offer medical advice to individuals. If you have specific concerns about your health or genetic testing, we suggest that you discuss them with your health care provider.****

## References

[ref-3161194526] U.S. Preventive Services Task Force. Screening for colorectal cancer: U.S. Preventive Services Task Force recommendation statement. Ann Intern Med. 2008 Nov 4;149(9):627-37. Epub 2008 Oct 6. 18838716 10.7326/0003-4819-149-9-200811040-00243

[ref-1179496296] Edwards BK, Ward E, Kohler BA, Eheman C, Zauber AG, Anderson RN, Jemal A, Schymura MJ, Lansdorp-Vogelaar I, Seeff LC, van Ballegooijen M, Goede SL, Ries LA. Annual report to the nation on the status of cancer, 1975-2006, featuring colorectal cancer trends and impact of interventions (risk factors, screening, and treatment) to reduce future rates. Cancer. 2010 Feb 1;116(3):544-73. 19998273 10.1002/cncr.24760 PMC3619726

[ref-2892742440] Altekruse, S.F., Kosary, C.L., Krapcho, M., Neyman, N., Aminou, R., Waldron, W., Ruhl, J., Howlader, N., Tatalovich, Z., Cho, H., Mariotto, A., Eisner, M.P., Lewis, D.R., Cronin, K., Chen, H.S., Feuer, E.J., Stinchcomb, D.G., Edwards, B.K. (eds). SEER Cancer Statistics Review, 1975-2007, National Cancer Institute (Bethesda, MD), based on November 2009 SEER data submission, posted to the SEER web site, 2010.http://seer.cancer.gov/csr/1975_2007/

[ref-1877619540] Henley SJ, King JB, German RR, Richardson LC, Plescia M; Centers for Disease Control and Prevention (CDC). Surveillance of screening-detected cancers (colon and rectum, breast, and cervix) - United States, 2004-2006. MMWR Surveill Summ. 2010 Nov 26;59(9):1-25. 21102407

[ref-3761240973] Whitlock EP, Lin J, Liles E, Beil T, Fu R, O'Connor E, Thompson RN, Cardenas T. Screening for Colorectal Cancer: An Updated Systematic Review [Internet]. Rockville (MD): Agency for Healthcare Research and Quality (US); 2008 Oct. Available from http://www.ncbi.nlm.nih.gov/bookshelf/br.fcgi?book=es65v1. 20722162

[ref-2288438972] Laboratory Corporation of America (LabCorp). Technical Review: ColoSure™, A Stool DNA-based Colorectal Cancer Screening Test (2008). Available athttps://www.labcorp.com/wps/portal/!ut/p/c1/hY1BDoIwEEXPwglmmNaxLtFg7cISUo20m6YLYzACLozntxwA_H_53s-HALlj-vaP9OmnMb2gg8CxVWxbsoTqTEckwXvcHOoSNWfulznin_Vt_lvfzxwXUiHY0zTcwUPYxmstXWl2AhvtCI0TjbRkmRXCBbzOkl-V3kOHT5miqYriB5Ii0gg!/dl2/d1/L0lJWXBwZyEhL3dIRUJGUUJnc2FBTUJyQ0svWUk1eWx3ISEvN19VRTRTMUk5MzBPR1MyMElTM080TjJONjY4MC92aWV3VGVzdA!!/?testId=1188079.

[ref-1034541974] American Cancer Society: All about Colon and Rectum cancer. Accessed on December 13, 2010.http://www.cancer.org/Cancer/ColonandRectumCancer/index.

[ref-589637597] Ahlquist DA. Molecular detection of colorectal neoplasia. Gastroenterology. 2010 Jun;138(6):2127-39. Review. 20420950 10.1053/j.gastro.2010.01.055

[ref-1905073336] Grady WM, Carethers JM. Genomic and epigenetic instability in colorectal cancer pathogenesis. Gastroenterology. 2008 Oct;135(4):1079-99. Epub 2008 Sep 4. Review. 18773902 10.1053/j.gastro.2008.07.076 PMC2866182

[ref-3346629510] Sinčić N, Herceg Z. DNA methylation and cancer: ghosts and angels above the genes. Curr Opin Oncol. 2011 Jan;23(1):69-76. 21119515 10.1097/CCO.0b013e3283412eb4

[ref-3963375559] Chen WD, Han ZJ, Skoletsky J, Olson J, Sah J, Myeroff L, Platzer P, Lu S, Dawson D, Willis J, Pretlow TP, Lutterbaugh J, Kasturi L, Willson JK, Rao JS, Shuber A, Markowitz SD. Detection in fecal DNA of colon cancer-specific methylation of the nonexpressed vimentin gene. J Natl Cancer Inst. 2005 Aug 3;97(15):1124-32. 16077070 10.1093/jnci/dji204

[ref-4083606815] Kann L, Han J, Ahlquist D, Levin T, Rex D, Whitney D, Markowitz S, Shuber A. Improved marker combination for detection of de novo genetic variation and aberrant DNA in colorectal neoplasia. Clin Chem. 2006 Dec;52(12):2299-302. Epub 2006 Nov 2. 17082247 10.1373/clinchem.2007.070896

[ref-296247484] Zou H, Harrington JJ, Shire AM, Rego RL, Wang L, Campbell ME, Oberg AL, Ahlquist DA. Highly methylated genes in colorectal neoplasia: implications for screening. Cancer Epidemiol Biomarkers Prev. 2007 Dec;16(12):2686-96. 18086775 10.1158/1055-9965.EPI-07-0518

[ref-3172305302] Ahlquist DA, Sargent DJ, Loprinzi CL, Levin TR, Rex DK, Ahnen DJ, Knigge K, Lance MP, Burgart LJ, Hamilton SR, Allison JE, Lawson MJ, Devens ME, Harrington JJ, Hillman SL. Stool DNA and occult blood testing for screen detection of colorectal neoplasia. Ann Intern Med. 2008 Oct 7;149(7):441-50, W81. 18838724 10.7326/0003-4819-149-7-200810070-00004 PMC4016975

[ref-3276069857] Shirahata A, Sakata M, Sakuraba K, Goto T, Mizukami H, Saito M, Ishibashi K, Kigawa G, Nemoto H, Sanada Y, Hibi K. Vimentin methylation as a marker for advanced colorectal carcinoma. Anticancer Res. 2009 Jan;29(1):279-81. 19331162

[ref-3997455750] Min, B., Cho, M., Lee, K. & Lee, J. Aberrant Promoter Methylation of Vimentin Gene in Colorecal Cancer Associated with Adenoma-Carcinoma Sequence. Ann Oncology 21 (Supplement 6), vi31-vi121, Abstract P-0299 (2010).

[ref-928360697] Laboratory Corporation of America (LabCorp). ColoSure™ Colorectal Cancer Detection. Accessed November 22, 2010.https://www.labcorp.com/wps/portal/!ut/p/c1/hY1BDoIwEEXPwglmmNaxLtFg7cISUo20m6YLYzACLozntxwA_H_53s-HALlj-vaP9OmnMb2gg8CxVWxbsoTqTEckwXvcHOoSNWfulznin_Vt_lvfzxwXUiHY0zTcwUPYxmstXWl2AhvtCI0TjbRkmRXCBbzOkl-V3kOHT5miqYriB5Ii0gg!/dl2/d1/L0lJWXBwZyEhL3dIRUJGUUJnc2FBTUJyQ0svWUk1eWx3ISEvN19VRTRTMUk5MzBPR1MyMElTM080TjJONjY4MC92aWV3VGVzdA!!/?testId=1188079.

[ref-2414545072] DNA Direct. ColoSure™ (Stool DNA Testing): Who Should Consider ColoSure™ Colon Cancer Screening? Accessed November 22, 2010.https://www.dnadirect.com/web/article/screening-tests/colosure-stool-dna-testing/24/who-should-consider-screening.

[ref-3018992414] DNA Direct. About the Genomic Medicine Institute Network. Accessed November 22, 2010.http://www.dnadirect.com/web/consumers/gmi-about.

[ref-2840348922] U.S. Cancer Statistics Working Group. United States Cancer Statistics: 1999–2007 Incidence and Mortality Web-based Report. Atlanta: U.S. Department of Health and Human Services, Centers for Disease Control and Prevention and National Cancer Institute; 2010. Available at:http://www.cdc.gov/uscs.

[ref-962919701] Bixquert-Jiménez M. Selective colorectal cancer screening in average-risk populations. Rev Esp Enferm Dig. 2009 Dec;101(12):821-9. English, Spanish. 20082542 10.4321/s1130-01082009001200001

[ref-34856068] Holden DJ, Jonas DE, Porterfield DS, Reuland D, Harris R. Systematic review: enhancing the use and quality of colorectal cancer screening. Ann Intern Med. 2010 May 18;152(10):668-76. Epub 2010 Apr 13. Review. 20388703 10.7326/0003-4819-152-10-201005180-00239

[ref-3729702293] Holden, DJ, Harris, R, Porterfield, DS, Jonas, DE, Morgan, LC, Reuland, D, Gilchrist, M, Viswanathan, M, Lohr, KN, Lyda-McDonald, B. Enhancing the Use and Quality of Colorectal Cancer Screening. Evidence Report/Technology Assessment No.190. (Prepared by the RTI International–University of North Carolina Evidence-based Practice Center under Contract No. 290-2007-10056-I.) AHRQ Publication No. 10-E-002. Rockville, MD: Agency for Healthcare Research and Quality. February 2010. PMC4781029 20726624

[ref-604939278] Whitlock EP, Lin JS, Liles E, Beil TL, Fu R. Screening for colorectal cancer: a targeted, updated systematic review for the U.S. Preventive Services Task Force. Ann Intern Med. 2008 Nov 4;149(9):638-58. Epub 2008 Oct 6. Review. 18838718 10.7326/0003-4819-149-9-200811040-00245

[ref-3143998740] Levin B, Lieberman DA, McFarland B, Smith RA, Brooks D, Andrews KS, Dash C, Giardiello FM, Glick S, Levin TR, Pickhardt P, Rex DK, Thorson A, Winawer SJ; American Cancer Society Colorectal Cancer Advisory Group; US Multi-Society Task Force; American College of Radiology Colon Cancer Committee. Screening and surveillance for the early detection of colorectal cancer and adenomatous polyps, 2008: a joint guideline from the American Cancer Society, the US Multi-Society Task Force on Colorectal Cancer, and the American College of Radiology. CA Cancer J Clin. 2008 May-Jun;58(3):130-60. Epub 2008 Mar 5. 18322143 10.3322/CA.2007.0018

[ref-2614045336] Rex DK, Johnson DA, Anderson JC, Schoenfeld PS, Burke CA, Inadomi JM; American College of Gastroenterology. American College of Gastroenterology guidelines for colorectal cancer screening 2009 [corrected]. Am J Gastroenterol. 2009 Mar;104(3):739-50. Epub 2009 Feb 24. Erratum in: Am J Gastroenterol. 2009 Jun;104(6):1613. 19240699 10.1038/ajg.2009.104

[ref-3692209180] American Academy of Family Physicians. Summary of Recommendations for Clinical Preventive Services, June 2010. Accessed December 7, 2010. (2010).http://www.aafp.org/online/en/home/clinical/exam.html.

[ref-2573917867] Burt RW, Barthel JS, Dunn KB, David DS, Drelichman E, Ford JM, Giardiello FM, Gruber SB, Halverson AL, Hamilton SR, Ismail MK, Jasperson K, Lazenby AJ, Lynch PM, Martin EW Jr, Mayer RJ, Ness RM, Provenzale D, Rao MS, Shike M, Steinbach G, Terdiman JP, Weinberg D; NCCN. NCCN clinical practice guidelines in oncology. Colorectal cancer screening. J Natl Compr Canc Netw. 2010 Jan;8(1):8-61. 20064289 10.6004/jnccn.2010.0003

[ref-130606179] Kaiser Permanente Care Management Institute. Colorectal cancer screening clinical practice guideline. Oakland (CA): Kaiser Permanente Care Management Institute; 2008 Dec. 190 p. [195 references].

[ref-4037625960] Aetna Clinical Policy Bulletin: Colorectal Cancer Screening. Accessed on December 15, 2010.http://www.aetna.com/cpb/medical/data/500_599/0516.html.

[ref-486672781] United Healthcare Fecal DNA Testing Policy. Accessed March 14, 2011.https://www.oxhp.com/secure/policy/fecal_dna_testing_311.html.

[ref-62902758] United Healthcare Colorectal Cancer Screening Policy. Accessed November 18, 2010.https://www.oxhp.com/secure/policy/colorectal_cancer_screening_1209.html.

[ref-2509175856] Li M, Chen WD, Papadopoulos N, Goodman SN, Bjerregaard NC, Laurberg S, Levin B, Juhl H, Arber N, Moinova H, Durkee K, Schmidt K, He Y, Diehl F, Velculescu VE, Zhou S, Diaz LA Jr, Kinzler KW, Markowitz SD, Vogelstein B. Sensitive digital quantification of DNA methylation in clinical samples. Nat Biotechnol. 2009 Sep;27(9):858-63. Epub 2009 Aug 16. PubMed Central PMCID: PMC2847606. 19684580 10.1038/nbt.1559 PMC2847606

[ref-575271589] Zou H, Harrington J, Rego RL, Ahlquist DA. A novel method to capture methylated human DNA from stool: implications for colorectal cancer screening. Clin Chem. 2007 Sep;53(9):1646-51. 17712002 10.1373/clinchem.2007.086223

[ref-2876894774] Loganayagam A. Faecal screening of colorectal cancer. Int J Clin Pract. 2008 Mar;62(3):454-9. Epub 2007 Sep 20. Review. 17887994 10.1111/j.1742-1241.2007.01532.x

[ref-3868430145] Baek YH, Chang E, Kim YJ, Kim BK, Sohn JH, Park DI. Stool methylation-specific polymerase chain reaction assay for the detection of colorectal neoplasia in Korean patients. Dis Colon Rectum. 2009 Aug;52(8):1452-9; discussion 1459-63. 19617759 10.1007/DCR.0b013e3181a79533

[ref-3505014464] Itzkowitz S, Brand R, Jandorf L, Durkee K, Millholland J, Rabeneck L, Schroy PC 3rd, Sontag S, Johnson D, Markowitz S, Paszat L, Berger BM. A simplified, noninvasive stool DNA test for colorectal cancer detection. Am J Gastroenterol. 2008 Nov;103(11):2862-70. Epub 2008 Aug 27. 18759824 10.1111/j.1572-0241.2008.02088.x

[ref-2959320311] Itzkowitz SH, Jandorf L, Brand R, Rabeneck L, Schroy PC 3rd, Sontag S, Johnson D, Skoletsky J, Durkee K, Markowitz S, Shuber A. Improved fecal DNA test for colorectal cancer screening. Clin Gastroenterol Hepatol. 2007 Jan;5(1):111-7. Epub 2006 Dec 8. 17161655 10.1016/j.cgh.2006.10.006

[ref-3756309907] Bossuyt PM. Clinical validity: defining biomarker performance. Scand J Clin Lab Invest Suppl. 2010 Jul;242:46-52. 20515277 10.3109/00365513.2010.493383

[ref-496142862] Lijmer JG, Mol BW, Heisterkamp S, Bonsel GJ, Prins MH, van der Meulen JH, Bossuyt PM. Empirical evidence of design-related bias in studies of diagnostic tests. JAMA. 1999 Sep 15;282(11):1061-6. Erratum in: JAMA 2000 Apr 19;283(15):1963. 10493205 10.1001/jama.282.11.1061

[ref-3663258644] Rutjes AW, Reitsma JB, Di Nisio M, Smidt N, van Rijn JC, Bossuyt PM. Evidence of bias and variation in diagnostic accuracy studies. CMAJ. 2006 Feb 14;174(4):469-76. 16477057 10.1503/cmaj.050090 PMC1373751

[ref-3885999594] Ahlquist, D., et al. Next Generation Stool DNA Testing for Detection of Colorectal Neoplasia: Early Marker Evaluation. Poster presentation at the 101st American Association for Cancer Research (AACR) Annual Meeting (2010). Available athttp://www.exactsciences.com/AACR%20poster_final-1.pdf.

[ref-4261643859] Imperiale TF, Ransohoff DF, Itzkowitz SH, Turnbull BA, Ross ME; Colorectal Cancer Study Group. Fecal DNA versus fecal occult blood for colorectal-cancer screening in an average-risk population. N Engl J Med. 2004 Dec 23;351(26):2704-14. 15616205 10.1056/NEJMoa033403

[ref-1605108720] Berger BM, Schroy PC 3rd, Rosenberg JL, Lai-Goldman M, Eisenberg M, Brown T, Rochelle RB, Billings PR. Colorectal cancer screening using stool DNA analysis in clinical practice: early clinical experience with respect to patient acceptance and colonoscopic follow-up of abnormal tests. Clin Colorectal Cancer. 2006 Jan;5(5):338-43. 16512992 10.3816/CCC.2006.n.003

[ref-3664805457] Schroy PC 3rd, Heeren TC. Patient perceptions of stool-based DNA testing for colorectal cancer screening. Am J Prev Med. 2005 Feb;28(2):208-14. 15710277 10.1016/j.amepre.2004.10.008

[ref-1829311724] Schroy PC 3rd, Lal S, Glick JT, Robinson PA, Zamor P, Heeren TC. Patient preferences for colorectal cancer screening: how does stool DNA testing fare? Am J Manag Care. 2007 Jul;13(7):393-400. 17620034

[ref-2991231684] Rosman AS, Korsten MA. Effect of verification bias on the sensitivity of fecal occult blood testing: a meta-analysis. J Gen Intern Med. 2010 Nov;25(11):1211-21. Epub 2010 May 25. Review. PubMed Central PMCID: PMC2947648. 20499198 10.1007/s11606-010-1375-0 PMC2947648

[ref-3355224051] Zauber, A.G., et al. Cost-Effectiveness of DNA Stool Testing to Screen for Colorectal Cancer. Agency for Healthcare Research and Quality (AHRQ) Technology Assessment. December 2007. Available athttps://www.cms.gov/determinationprocess/downloads/id52TA.pdf. 25879132

[ref-3223163935] Fenton JJ, Elmore JG, Buist DS, Reid RJ, Tancredi DJ, Baldwin LM. Longitudinal adherence with fecal occult blood test screening in community practice. Ann Fam Med. 2010 Sep-Oct;8(5):397-401. PubMed Central PMCID: PMC2939414. 20843880 10.1370/afm.1133 PMC2939414

[ref-4049540477] Genomeweb. Exact Sciences' Shares Take a Hit Following Colorectal Cancer Dx Presentation. Accessed November 1, 2010.http://www.genomeweb.com/dxpgx/exact-sciences-shares-take-hit-following-colorectal-cancer-dx-presentation.

[ref-3722109558] Exact Sciences Corporation. Exact Sciences Investor Update October 28, 2010. Accessed November 1, 2010. Available fromhttp://www.exactsciences.com/.

